# Novel capsid binder and PI4KIIIbeta inhibitors for EV-A71 replication inhibition

**DOI:** 10.1038/s41598-021-89271-8

**Published:** 2021-05-06

**Authors:** Yong Wah Tan, Wan Keat Yam, Rachel Jia Wen Kooi, Jacob Westman, Gustav Arbrandt, Justin Jang Hann Chu

**Affiliations:** 1 Collaborative and Translation Unit for HFMD, Institute of Molecular and Cell Biology, Agency for Science, Technology and Research (A*STAR), Singapore, Singapore; 2 Curovir AB, Stockholm, Sweden; 3 Laboratory of Molecular RNA Virology and Antiviral Strategies, Department of Microbiology and Immunology, Yong Loo Lin School of Medicine, National University of Singapore, Singapore, Singapore

**Keywords:** Virology, Antivirals

## Abstract

The Hand, Foot and Mouth Disease (HFMD) is a highly contagious viral illness generally manifests as a mild disease in young children and immunocompromised adults. It has however emerged as a significant public health threat in recent years as outbreaks have been occurring regularly, especially in the Asia–Pacific. The disease can result from infections by a wide variety of human enteroviruses, particularly, Enterovirus A71 (EV-A71) has garnered more attention due to its association with severe disease in infected patients. Despite the potential to result severe neurological complications or even fatality, there is currently no effective antiviral for treatment of EV-A71 infections and the only vaccines available are restricted to distribution in China. In this study, we report the in vitro and in vivo evaluation of two candidate antiviral compounds active against EV-A71, a viral capsid inhibitor (G197) and a novel host-targeting phosphatidylinositol 4-kinase III beta inhibitor (N373) which, especially when used in combination, can significantly improve the survival and pathology of infected mice.

## Introduction

Enterovirus A71 (EV-A71) is a highly infectious pathogen from the *Picornaviridae* family of viruses and a clinically important aetiological agent of the Hand, Foot and Mouth Disease (HFMD), being frequently associated with cases of severe disease and fatality. HFMD can result from infections by a large number of enterovirus serotypes spanning two species, *Enterovirus A* and *Enterovirus B*^1–3^, with prevailing serotypes changing temporally and spatially. Currently, there is no antiviral on the market for treatment of infections and the only vaccines available are restricted to distribution in China, leaving only supportive treatments available for afflicted patients. In the continued absence of a widely available vaccine, development of antivirals is crucial. Antiviral strategies for drug development against EV-A71 encompass both viral and host targeting molecules and viral capsid inhibitor has been an area of intensive research with several molecules identified to bind EV-A71 capsid proteins^4–8^, notably the extensively studied like vapendavir/BTA798^9^ and pleconaril^10^ which were originally developed for human rhinoviruses (HRV), the latter however has been confirmed to be inactive against EV-A71^11^. In addition, several other classes of compounds were also reported to possess EV-A71 capsid binding activities like pyridyl imidazolidinones^4,12,13^, aminopyridyl 1,2,5-thiadiazolidine 1,1-dioxides^7^ and a sulfonated food azo dye, Brilliant Black BN^14^.

Host protein phosphatidylinositol 4-kinase III beta (PI4KIIIβ) has been shown to play important roles in the replication of several virus families, including *Picornaviridae*^15–17^, hepatitis C virus (HCV) of *Flaviviridae*^18^ and severe acute respiratory syndrome coronavirus (SARS CoV)^19^. Specifically for enteroviruses, PI4KIIIβ is actively recruited from the Golgi to the replication organelles^20,21^ and the product of its kinase activity, phosphatidylinositol 4-phosphate (PI4P), is essential for virus replication^22^. Inhibitors of PI4KIIIβ have demonstrated excellent in vitro inhibition of many picornaviruses^21,22^ although some were found to exhibit high toxicities in vivo^23^.

Despite rigorous research into antivirals for EV-A71 and other enteroviruses, none had been approved for use due to limited efficacy or safety concerns^24^, driving the need to develop new antivirals with low host toxicities. Furthermore, RNA viruses like EV-A71 mutate at very high frequencies, allowing them to adapt to single compound treatments easily. This presents a case for a combination therapy approach to developing antiviral treatments. As a collaboration with Curovir which specializes in the development of novel antivirals, we evaluated the in vitro and in vivo efficacy of a capsid binding inhibitor for EV-A71, G197, which has demonstrated in vitro efficacy superior to BTA-798. In addition, we have also assessed a panel of novel PI4KIIIβ inhibitors developed by Curovir for antiviral efficacy both in vitro followed by the shortlisting of N373 for further studies both in vitro and in vivo.

## Results

### Post-infection treatment with G197 and N373 inhibits EV-A71 replication in vitro

**Figure 1:**
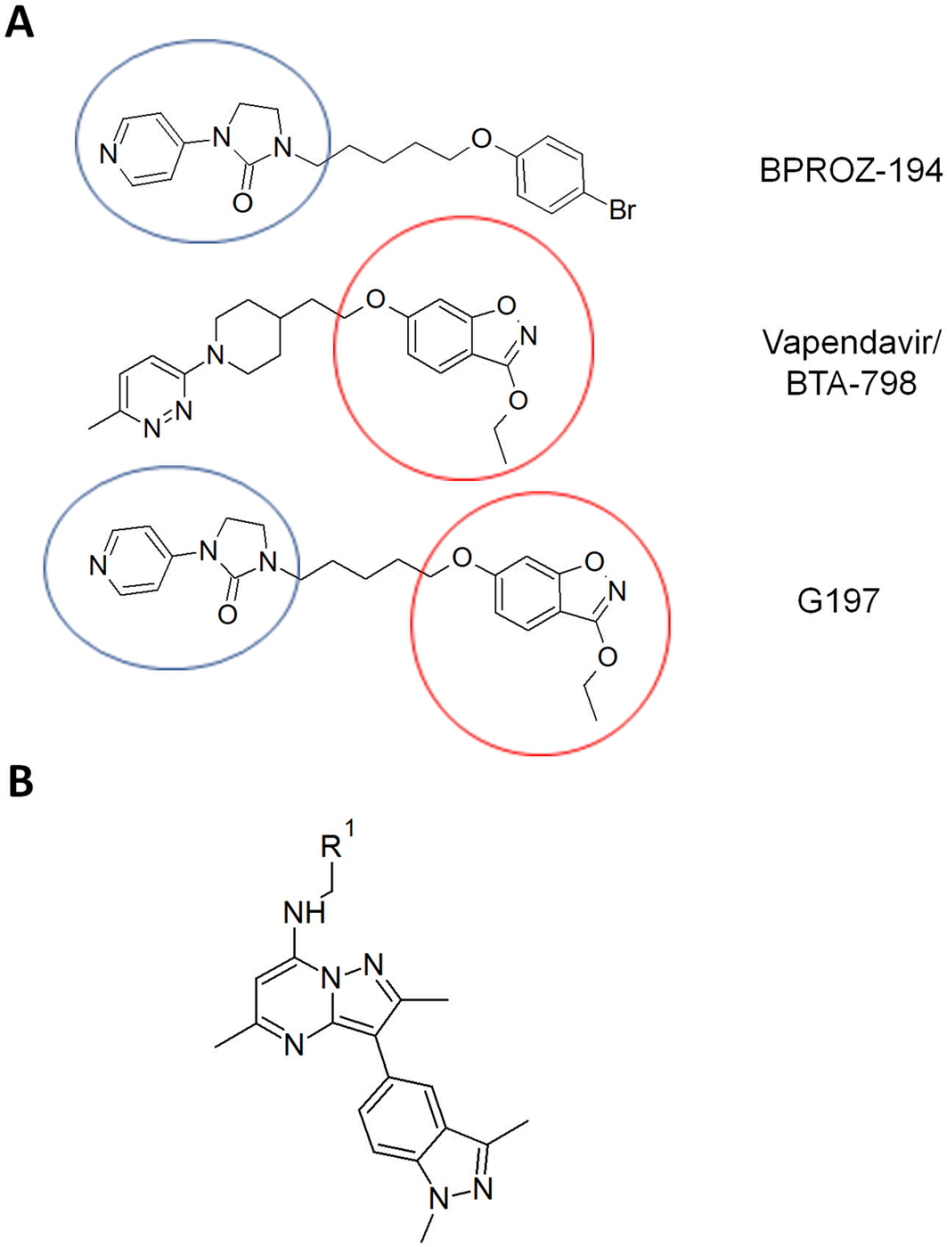
Chemical structures of G197 and N373. (**A**) G197 is a structural chimera of a capsid inhibitor reported by Shia et al.^13^ (blue circle) and vapendavir (BTA-798) (red circle). (**B**) The general structure of N373 is as shown and R^1^ represents the unique chemical group that is covered by patents owned by Curovir.

**Figure 2:**
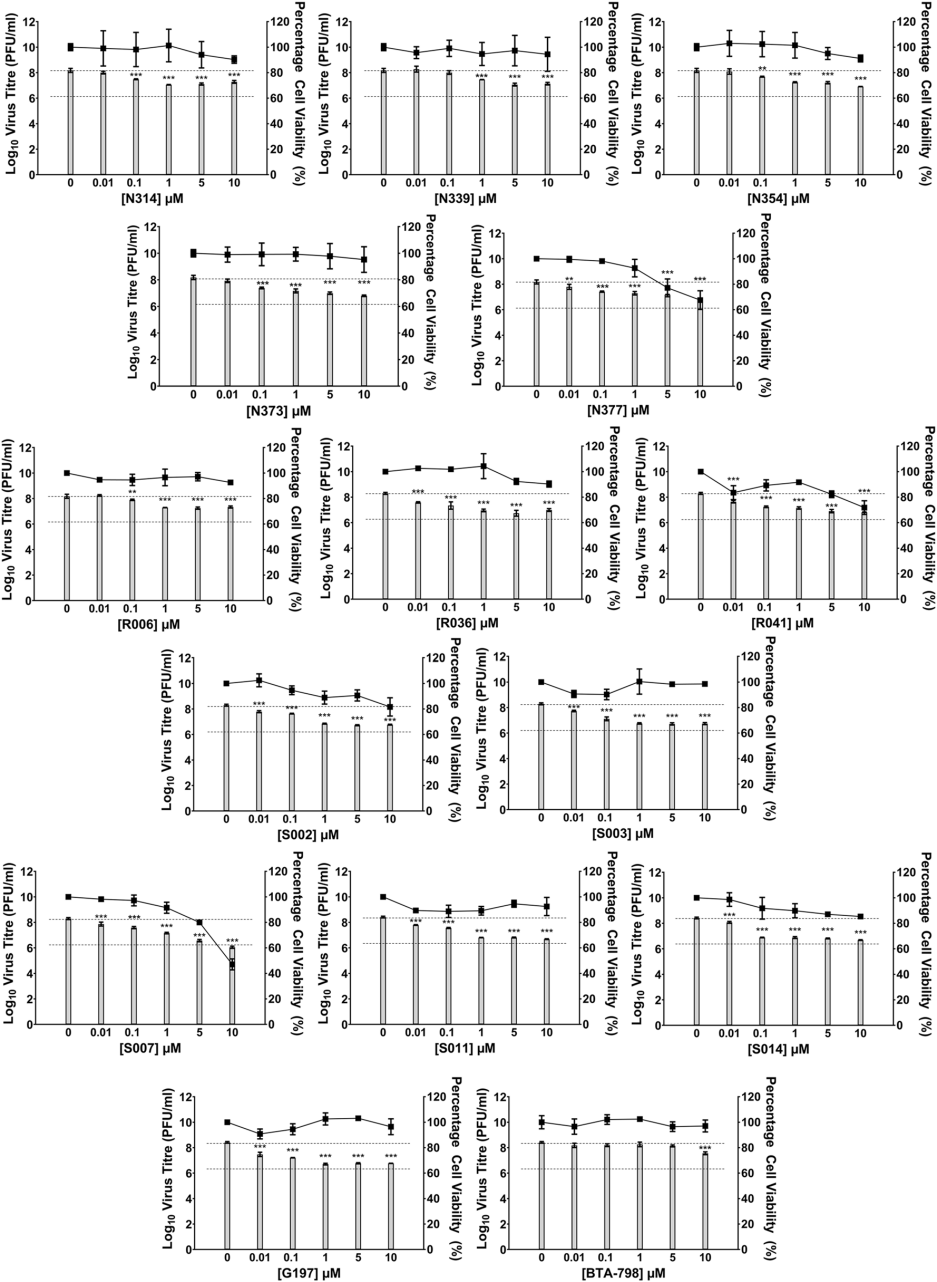
Effect of novel PI4KIIIβ and capsid inhibitors on the replication of EV-A71 in vitro. RD cells infected with EV-A71 was treated with each of the compounds at 5 different concentrations and 0 µM refers to control infected cells treated with 0.1% DMSO. Virus titres achieved at 12 h.p.i. was determined by viral plaque assays and represented as bars on each of the charts. Cell viability of drug-treated non-infected cells was assessed by alamarBLUE Cell Viability Reagent and expressed as a percentage compared to control DMSO-treated cells. Novel PI4KIIIβ inhibitors were: N314, N339, N354, N373, N377, R006, R036, R041, S002, S003, S007, S011 and S014. N373 was selected for further studies based on superior cell viability profile and inhibition of EV-A71 replication. G197 was found to be more effective in inhibiting EV-A71 replication than control capsid inhibitor BTA-798. Bars represent Log_10_ virus titres (PFU/ml) and points represent cell viability across the different compound concentrations. Dashed lines indicate negative control virus titre (top line) and a 2 Log_10_ unit reduction in virus titre (lower line). Statistical analysis for differences in virus titre due to drug treatment was performed with one-way ANOVA with Dunnett’s post-test: *(*p* < 0.05), **(*p* < 0.01), ***(*p* < 0.005).

To identify a candidate PI4KIIIβ inhibitor, a total of 13 synthesized compounds were evaluated together with candidate capsid inhibitor (G197) for in vitro antiviral activity against EV-A71 in RD cells. G197 was designed to be a structural chimera of an EV-A71 capsid inhibitor (BPROZ-194) reported by Shia et al.^13^ and vapendavir/BTA-798 (Fig. 1A). RD cells infected with EV-A71 (MOI = 1) were treated with each of the compounds at different concentrations and virus titres were determined by viral plaque assay at 12 h post-infection (h.p.i.) (post-treatment studies). To determine the impact of compound treatments on cell viability, non-infected RD cells were treated with each of the compounds at indicated concentrations for 12 h and cell viability was determined by alamarBlue Cell Viability Assay (Thermo Fisher Scientific). The results from both assays were presented together in Fig. 2. For capsid inhibitors, minimal reduction in cell viability was observed over the range of concentrations tested (> 90% viability) and G197 demonstrated higher antiviral activity against EV-A71, reducing viral titre by at 1 Log_10_ unit at 1 µM, compared to vapendavir/BTA-798, which exhibited some activity (approximate reduction of 0.5 Log_10_ unit) at 10 µM.

**Figure 3:**
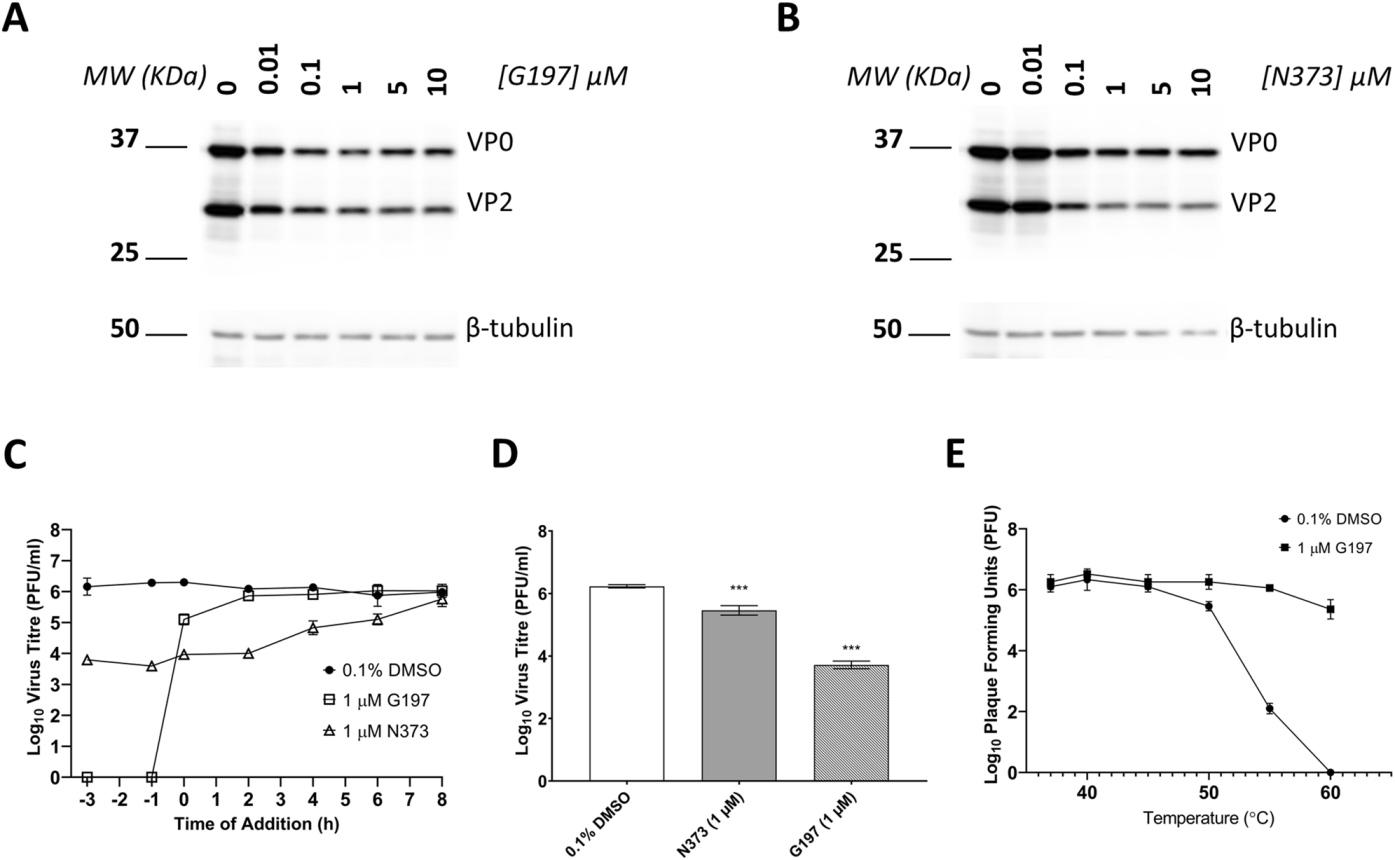
Treatment with G197 and N373 resulted decreased viral protein translation and treatment with G197 before virus entry resulted in greater inhibition efficacy consistent with the behaviour of capsid binding inhibitors. (**A**) Post-treatment of infected cells with G197 and (**B**) N373 resulted in a decrease in VP2 and precursor VP0 expression. Full length blots available were included as Supplementary Information 1. (**C**) Time-of-addition assay with G197 and N373 at 1 µM and negative control 0.1% DMSO. Treatment of cells with G197 before 0 h result in an abolishment of virus replication and addition of after virus has entered the cells were only able to reduce virus titres by a maximum of 1 Log_10_ unit and no inhibition was observed from 2 h.p.i. N373 treatment gradually lost efficacy with delayed treatment up to 2 h.p.i. to no inhibition at 8 h.p.i. (**D**) Treatment of cells with G197 and N373 during the 1 h virus inoculation reduced virus titres significantly and G197 was more effective than N373. Statistical analysis for differences in virus titre due to drug treatment was performed with one-way ANOVA with Dunnett’s post-test: ***(*p* < 0.005). (**E**) Thermal stability of EV-A71 was assessed by incubating virus suspensions at temperatures from 37 to 60 °C in the presence of 0.1% DMSO or 1 µM G197. The presence of G197 improved the thermal stability of virus particles at elevated temperatures.

Among the PI4KIIIβ inhibitors, 11 compounds (except N339 and R036) were found to reduce EV-A71 titre by at least 0.5 Log_10_ unit at 0.1 µM (Fig. 2), the primary criteria for antiviral activity. Cell viability profiles of the compounds were compared and N373 was shortlisted for further evaluation together with capsid inhibitor G197 due to its superior cell viability profile that was maintained above 95% across all concentrations tested. The basic structure of N373 is presented in Fig. 1B. Western blot was performed on lysates from infected cells treated with G197 (Fig. 3A) or N373 (Fig. 3B) and a dose-dependent decrease in viral protein VP2 (and its precursor VP0) was observed up to 1 µM, beyond which viral protein expression did not appear to be significantly inhibited by the higher drug concentrations. This result indicated the reduction in viral protein expression upon treatment with G197 and N373 contributed to the drop in virus titres observed in Fig. 2.

### N373 inhibits the activity of PI4KIIIβ in vitro and G197 is a capsid-binding inhibitor of EV-A71

To confirm the in vitro activity and specificity of N373, ADP-Glo Kinase Assay (Promega) was performed with recombinant PI4KIIIα and PI4KIIIβ. N373 demonstrated greater inhibition efficacy against PI4KIIIβ than PI4KIIIα (Supplementary Fig. S1), confirming its in vitro activity as a specific PI4KIIIβ inhibitor. To confirm the function of G197 as a capsid binding inhibitor of EV-A71, a time-of-addition assay was performed using 1 µM of G197, N373 and 0.1% DMSO negative control were added at different time-points before and after virus inoculation (MOI = 1) and addition of G197 post-inoculation (from 0 h.p.i.) was much less effective compared to addition prior to virus inoculation (Fig. 3C) with no virus detected at 12 h.p.i. when G197 was added from 2 h.p.i. In contrast, the addition of N373 prior to virus inoculation did not result in further inhibition of virus replication from addition at 0 h.p.i. and its efficacy was lowered when added to the infected cells from 4 h.p.i. Negative control, 0.1% DMSO, was consistent through all time-points of addition.

Next, we assessed the inhibition efficacy of the G197 and N373 during virus inoculation by incubating the virus with the compounds together with for 1 h followed by removal of both virus and compounds and the infected cells were cultured in media containing 2% FBS until 12 h.p.i. Treatment of cells with 1 µM G197 resulted in a 2 Log_10_ unit reduction of virus titre compared to negative control (0.1% DMSO), more potent than N373 which only resulted in less than 1 Log_10_ unit virus titre reduction in virus titre (Fig. 3D). As capsid binding inhibitors typically stabilize the capsid structure, we assessed the thermal stability of EV-A71 by viral plaque assay. Virus suspensions were incubated for 2 min at indicated temperatures between 37 and 60 °C in the presence of 1 µM G197 or 0.1% DMSO then titrated immediately. The titre of virus suspensions incubated with G197 stayed consistent up to 55 °C with a slight drop observed at 60 °C (Fig. 3E). In contrast, control virus suspension titres dropped by 0.5 Log_10_ unit at 50 °C followed by a sharp decline to undetectable levels at 60 °C. The presence of G197 increased the thermal stability of virus particles compared to DMSO control, consistent with the properties of capsid binding inhibitors. With these results (Fig. 3), we were able to conclude that G197 is a capsid-binding inhibitor of EV-A71 and N373 is a host-acting inhibitor that inhibits the kinase activity of PI4KIIIβ.

### Post-inoculation treatment with both N373 and G197 resulted in enhanced inhibition of EV-A71 replication

Since the two compounds have different mechanisms of action, we also explored the synergistic potential of the two compounds and double drug treatment was assessed in a post-treatment assay at two drug concentrations, 0.1 and 1 µM, and at MOIs 1 and 0.1, the latter was introduced to assessed probable impact on secondary infections in vitro.

**Figure 4:**
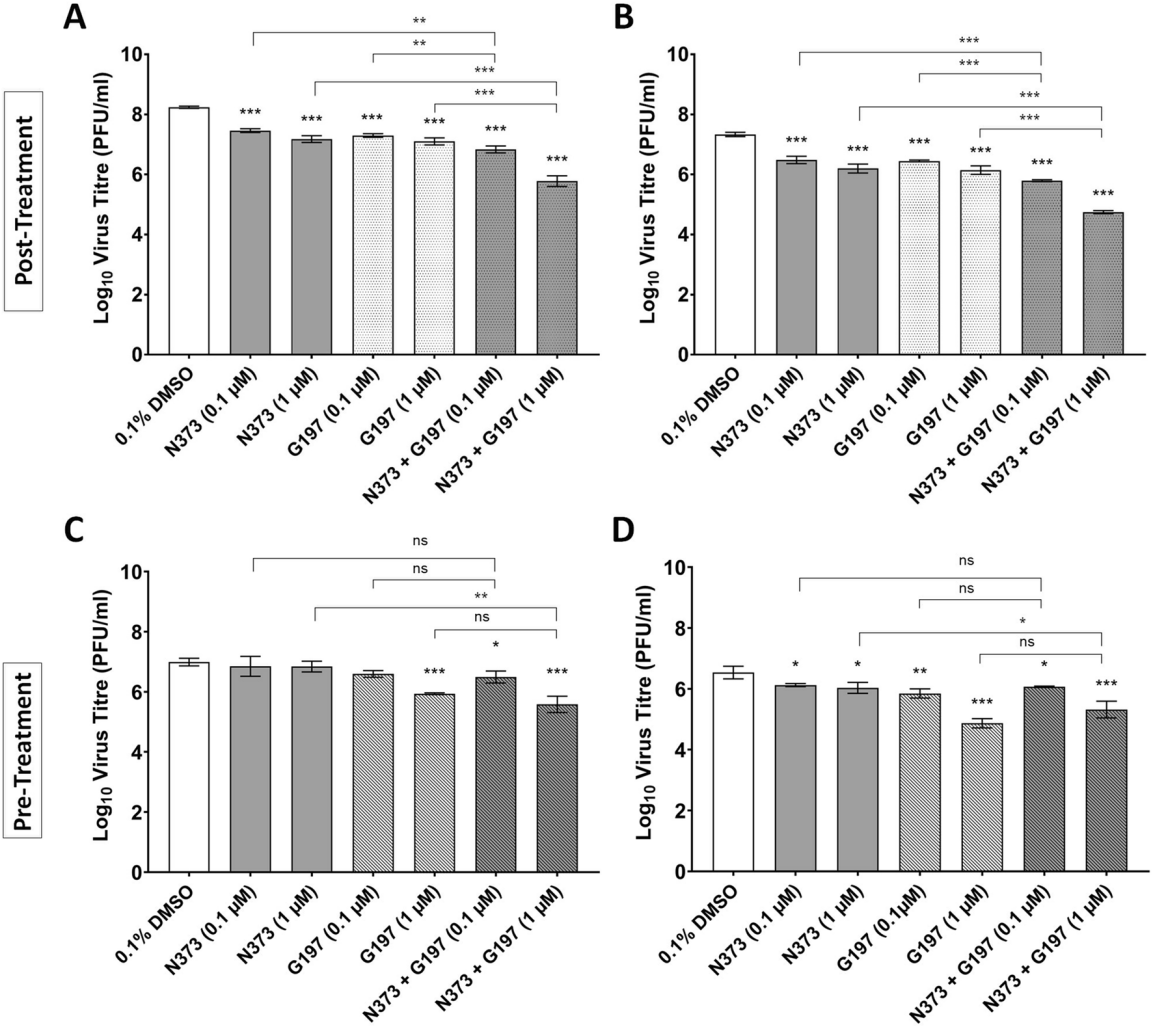
Treatment with both N373 and G197 exhibited greater inhibition of EV-A71 replication compared to single compounds when cells were treated after virus inoculation and not before. RD cells treated with N373 and G197 at the indicated combinations and concentrations were infected with EV-A71 after compound removal. Combination treatment (N373 + G197) at both concentrations resulted in virus titres lower than either of the single compound treatments at both MOI = 1 (**A**) and MOI = 0.1 (**B**). (**C**) RD cells were pre-treated for 2 h with N373 and G197 at the indicated combinations and concentrations prior to infection at MOI = 1 and (**D**) MOI = 0.1. Combination treatment did not result in a significantly lower virus titre compared to treatment with G197 only and N373 slightly reduced virus titres at a lower MOI. Statistical analysis for differences in virus titre due to drug treatment was performed with one-way ANOVA with Dunnett’s post-test: *(*p* < 0.05), **(*p* < 0.01), ***(*p* < 0.005). Student’s t-test was performed to compare single compound treatments (G197 or N373) to combination treatment at the same concentration.

At both MOIs tested, double compound treatments (N373 + G197) reduced EV-A71 titres to greater extents compared to single compound treatments. Specifically, at MOI 1, single compound treatment with N373 at 0.1 and 1 µM reduced virus titre achieved by approximately 0.8 and 1.1 Log_10_ unit respectively and a reduction by 0.9 and 1.1 Log_10_ unit was seen for G197 treated cells. Combination treatment with both compounds at 0.1 and 1 µM reduced the virus titres achieved by 1.4 and 2.5 Log_10_ unit respectively (Fig. 4A). At MOI = 0.1, treatment with only N373 at 0.1 and 1 µM reduced virus titres by 0.85 and 1.1 Log_10_ unit respectively and G197 treatment reduced the titres by 0.9 and 1.2 Log_10_ unit. When used in combination, virus titres achieved were reduced by approximately 1.5 and 2.6 Log_10_ unit at 0.1 and 1 µM respectively (Fig. 4B). In conclusion, N373 and G197 exhibited enhanced antiviral activity when RD cells were treated with both compounds compared to single compound treatments.

Since G197 is a capsid inhibitor, pre-treatment of cells with the compound was also performed and cells were treated 2 h prior to infection when the compounds are removed. We found that pre-treatment with G197 was inhibitory on EV-A71 replication at 1 µM at both MOI = 1 and 0.1 (Fig. 4C,D), reducing virus titres by 1 and 1.2 Log_10_ unit respectively. N373 slightly inhibited virus replication at 1 µM (MOI = 0.1) (Fig. 4D) and did not significantly affect the activity of G197 when used in combination.

### Administration of G197 and N373 improved survival of mice in a lethal EV-A71 challenge

Since the in vitro cell-based studies on N373 and G197 showed clear although somewhat limited antiviral activities as individual compounds and possible synergism between the two compounds, the compounds were evaluated in single compound and combination treatment regimens using a murine model of EV-A71 infection based on the same virus strain.

**Figure 5:**
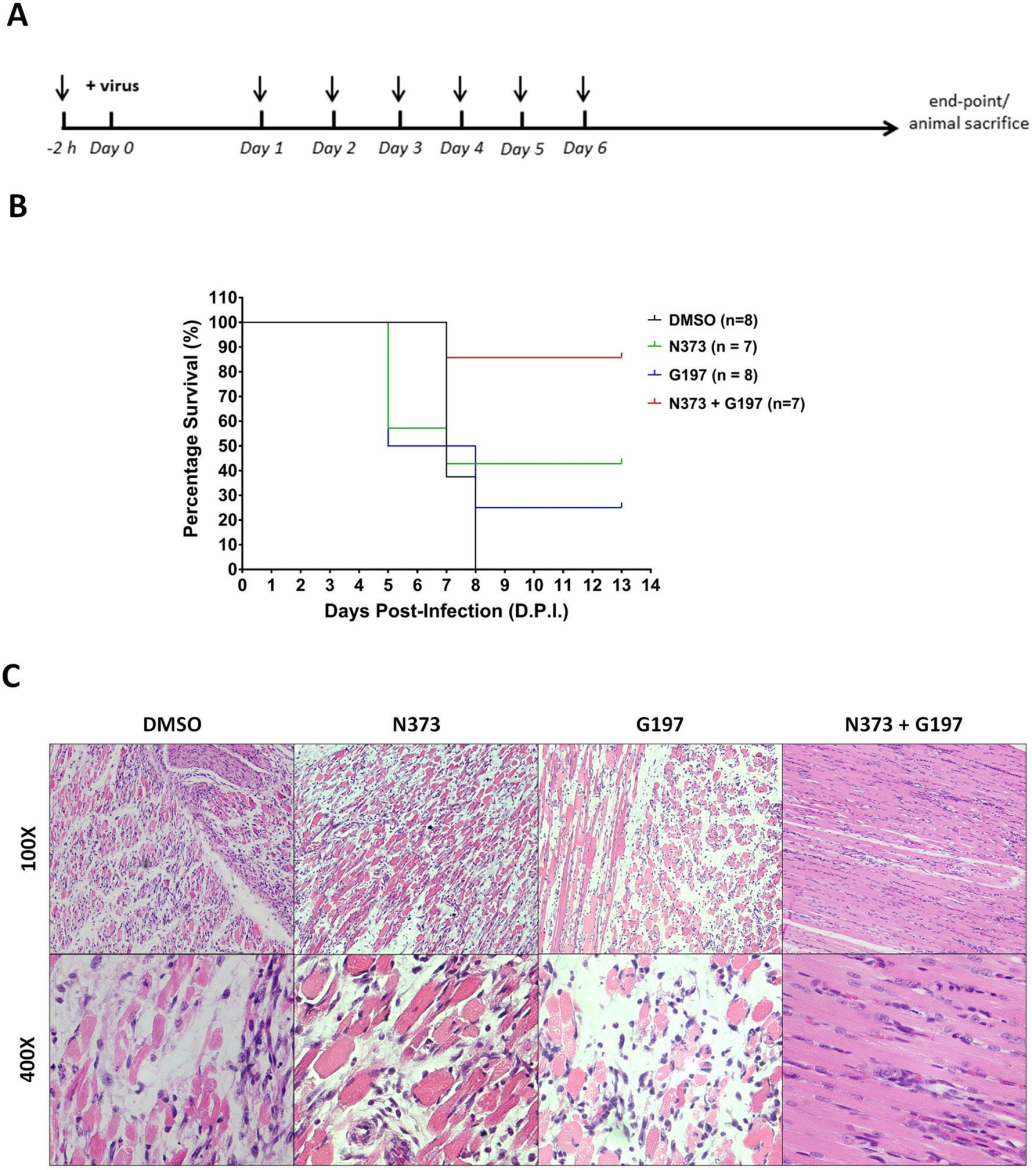
Treatment with both N373 and G197 improved survival and reduced muscle tissue pathology in EV-A71 infected mice. (**A**) Treatment regimen composed of a single dose 2 h prior to infection and 6 daily doses after infection. Animals were observed and sacrificed at 14 days post-infection for survival group and at 5 days post-infection for tissue harvesting group. (**B**) Survival of EV-A71 infected Balb/c neonates showed the greatest improvement when treated with both N373 and G197 followed by N373 only and a slight improvement with only G197 compared to control group (DMSO). (**C**) Haemotoxylin and eosin staining on harvested limb tissue of infected mice at 5 days post-infection showed breakdown of muscle tissue ranging from extensive in control group to negligible in combination treatment group (N373 + G197).

6-day old Balb/c neonates were lethally challenged with EV-A71, a single dose at 2 × 10^7^ plaque forming unit (PFU) and the treatments were carried out via intraperitoneal (i.p.) injections, as described in Fig. 5A with 0.1 ml/kg DMSO (control group), N373 (1 mg/kg) G197 (1 mg/kg), N373 + G197 (1 mg/kg each) (combi group). All animals were treated once at 2 h before virus inoculation (dose 1) followed by once daily for 6 consecutive days (dose 2–7). All animals were observed daily for clinical symptom presentation from the point of infection to 14 days post-infection (DPI). There were no survivors in DMSO control group (0% survival) and combination treatment (N373 + G197) group exhibited the greatest improvement at 85.7% survival. Single compound treatment (N373 and G197) groups exhibited survival rates of 42.9% and 25% respectively (Fig. 5B).

### Administration of G197 and N373 reduced muscle tissue pathology of EV-A71 infected mice

Examination of hind limb tissues from infected animals at 5 DPI yielded results consistent with the survival studies as the animals from the combination treatment (N373 + G197) group displayed significantly lesser muscular pathology compared to DMSO control treatment group which presented with extensive breakdown of muscle tissue (Fig. 5C). Tissues from single compound N373 and G197 treatment groups revealed slightly improved tissue integrity compared to DMSO control group. In conclusion, the results from the murine model of infection indicate in vivo antiviral efficacy of both N373 and G197 and using both compounds together can further improve the outcome of infection, evident in the markedly higher survival of animals in the combination treatment group.

### G197 and N373 inhibited replication of other serotypes of enteroviruses in vitro

In addition to in vivo studies of the two candidate antiviral compounds N373 and G197, we also explored their potential for broad spectrum activity against other HFMD-causing enteroviruses, especially for N373 which inhibits a host target. For this part of the study, antiviral activity of the two compounds used alone and in combination was assessed with two other *Enterovirus A* viruses, Coxsackievirus A6 (CV-A6) and A16 (CV-A16) as well as an *Enterovirus B* virus, Echovirus 7 (E-7) at MOI = 1.

**Figure 6:**
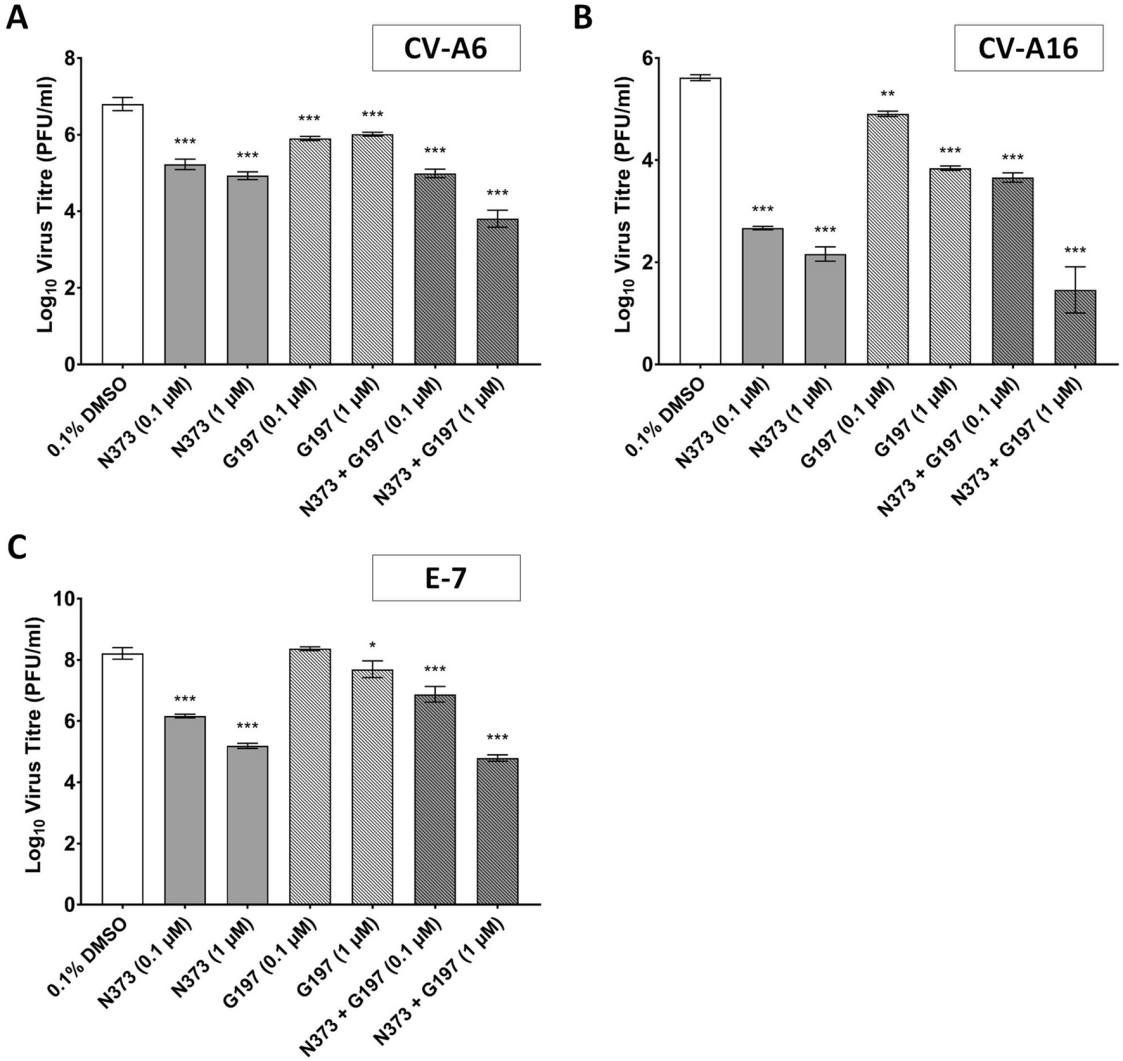
N373 and G197 inhibited virus replication of CV-A6, CV-A16 and E-7 to varying extents in vitro. RD cells infected at MOI = 1 with CV-A6, CV-A16 and E-7 were treated with compounds N373 and G197 at indicated combinations and concentrations. Virus supernatants harvested at experimental end-point were assessed by viral plaque assays to determine impact of compound treatments on virus replication. (**A**) Both N373 and G197 efficiently inhibited the replication of CV-A6 and (**B**) CV-A16. (**C**) N373 was able to efficiently inhibit E-7 replication in RD cells and G197 was only able to inhibit virus replication at the higher concentration of 1 µM. Statistical analysis for differences in virus titre due to drug treatment was performed with one-way ANOVA with Dunnett’s post-test: *(*p* < 0.05), **(*p* < 0.01), ***(*p* < 0.005).

In CV-A6 infected cells, treatment with N373 or G197 or in combination were effective in reducing virus titres at both concentrations tested and the combination appears to be at least as effective (0.1 µM), if not more effective (1 µM) than any single compound treatment (Fig. 6A). Specifically, at 0.1 µM, single compound treatments with N373 and G197 reduced the titres achieved by 1.5 and 0.9 Log_10_ unit respectively and combination treatment reduced the titre by 1.8 Log_10_ unit. At 1 µM, N373 was more effective and reduced the titre by 1.9 Log_10_ unit but no further reduction was noted for G197 with the increased concentration. Combination treatment at 1 µM showed an even stronger inhibition on CV-A6 replication, surpassing both single compound treatments, and reduced the virus titre by 3 Log_10_ unit.

In CV-A16 infected cells, while single compound with N373 or G197 and combination treatments were effective compared to DMSO control at both concentrations (Fig. 6B). N373 particularly effective against CV-A16, reducing virus titres by almost 3 and 3.5 Log_10_ unit at 0.1 and 1 µM respectively. The inhibition capacity of G197 was less pronounced at 0.7 (0.1 µM) and 1.7 (1 µM) Log_10_ unit. In cells treated with both compounds, virus titres were reduced by 2 (0.1 µM) and 4.2 (1 µM) Log_10_ unit.

For E-7 infected cells, only N373 was found to be inhibitory at both concentrations, reducing virus titres by 2 (0.1 µM) and 3 (1 µM) Log_10_ unit. G197 on the other hand only reduced the virus titre achieved by 0.5 Log_10_ unit at 1 µM and showed no inhibition at 0.1 µM (Fig. 6C). Combination treatment of E-7 infected cells at 0.1 µM was less effective than treatment with N373 alone having only reduced the virus titre by 1.3 Log_10_ unit. At 1 µM, combination treatment was slightly more effective than treatment with N373 with a reduction of virus titre by 3.4 Log_10_ unit.

N373 was found to be highly active against all three enteroviruses and more effective than G197 at reducing enterovirus titres in vitro, characteristic of a host-targeting mechanism, supporting its function as an inhibitor of PI4KIIIβ essential for its antiviral activities. G197 on the otherhand appears to be more active against *Enterovirus A* species as very little inhibition in virus replication was noted in E-7 infected cells.

## Discussion

In this study, we have identified two compounds, N373 and G197, as candidates for treatment of EV-A71 infections with minimal cellular toxicity exhibited at the concentrations tested. We have also tested these compounds at concentrations greater than 10 µM and found that only N373 was able to demonstrate increased inhibition of EV-A71 infection in cultured cells but the inhibition of virus replication did not further improve with higher concentrations of G197 (Supplementary Fig. S2). G197 was designed as a structural chimera of two enterovirus capsid inhibitors, BPROZ-194 and BTA-798, hence it was expected to inhibit viral replication with a similar mechanism. The time-of-addition and co-treatment assays performed in this study demonstrated the need for G197 to be present early or before infection to exert an effective antiviral activity and the thermal stability of EV-A71 in the presence of G197 supports the conclusion that G197 is indeed a capsid-binding inhibitor of EV-A71. In a bid to confirm the mechanism by which N373 could inhibit EV-A71 replication, in vitro activity of N373 was evaluated with ADP-Glo kinase assay for activity and N373 was found to be highly active against PI4KIIIβ and much less effective against PI4KIIIα (Supplementary Fig. S1). In addition, a less potent analogue was evaluated in place of N373 for the generation of resistance mutants due to difficulties obtaining a suboptimal concentration of N373 for this experiment. After 20 passages, a 10-times increase in IC_50_ but no total resistance to the compound was observed (unpublished data). Further analysis of the resistant mutants revealed mutations in viral protein 3A previously reported in CV-B3 resistant mutants to another PI4KIIIβ inhibitor^22^. Taken together, these information lend credence to N373 being a selective PI4KIIIβ inhibitor and was the mechanism by which it exerts its antiviral activity on the enteroviruses tested.

Results obtained from our in vitro studies showed somewhat limited but clear antiviral activities of both compounds against several *Enterovirus A* viruses when used individually. N373 also demonstrated effective inhibition on E-7 (*Enterovirus B*) while G197 was found to be much less effective. This result is not unexpected as G197, being a capsid binding compound, is expected to be more specific for closely related virus strains, i.e. within *Enterovirus A* species, rather than being efficient in binding to *Enterovirus B* and other *Enterovirus* species based on prior studies on capsid binding inhibitors^11,13,25^. N373 being a host targeting compound, has a greater potential for broad-spectrum antiviral activity against other species^26,27^. G197 on the otherhand, has the potential as an *Enterovirus A* specific antiviral compound.

While antiviral activities of N373 and G197 were not outstanding on their own, using them in combination proved to be much more effective in vitro. The in vivo studies also demonstrated that combination treatment was more effective in promoting the survival of EV-A71 infected mice as well as reducing the pathology in limb muscle tissue. Due to their high mutation rates, rapid development of drug resistance in enteroviruses is a major issue and is very well documented for capsid binders^6,28^ as well as host-targeting compounds^29–31^. Hence, combination usage of antivirals has the benefit of reducing the dosage of individual drugs, reducing host toxicity, and also potentially slow down the emergence of antiviral resistance.

The use of combination therapy regimes in the treatment of viral infection is not unheard of and have already been in use for several chronic viral infections like HCV and human immunodeficiency virus (HIV) infections^32–35^. Combination therapy has also been explored for EV-A71 with promising results in vitro^36^. A potential drawback of combination therapy is the added complexity in clinical trial design in addition to the potential for drug antagonism and side-effect synergism in humans. These issues however could potentially be minimized with a rigorous assessment of individual drug profiles prior to conducting trials for combination therapy. With rising awareness and reports of drug resistance development, including capsid inhibitors and even host-targeting compounds, combination therapy would likely become increasingly common and be the preferred type of treatment for infections in the future. Lastly, as G197 and N373 were only tested for in vivo efficacy against EV-A71, further studies with murine infection models for CV-A6, CV-A16 and E-7 will be required to validate the in vitro results on potential broad-spectrum antiviral activities of N373 and G197.

## Methods

### Ethics declaration

All experiments with the murine model of EV-A71 infection were performed with approval from the A*STAR Institutional Animal Care and Use Committee (IACUC) in accordance of the (Singapore) National Advisory Committee for Laboratory Animal Research (NACLAR). All methods utilized in this study were performed in compliance to ARRIVE Guidelines.

### Cells and viruses

EV-A71 (5865/SIN/00009), CV-A6 (laboratory strain), CV-A16 (G-10) and E-7 (Wallace) were propagated in Rhabdomyosarcoma (RD) cells (ATCC CCL-136). RD cells were maintained in Dulbecco’s modified Eagle’s medium/Ham’s F-12 (1:1) (DMEM/F-12) supplemented with 10% foetal bovine serum (FBS). For infection of cells, monolayers of RD cells were incubated with viruses suspended in DMEM/F-12 supplemented with 2% FBS for 1 h at 37 °C. Following removal of virus, cells were washed with phosphate-buffered saline (PBS) and fresh DMEM/F-12 + 2% FBS was added and the cells were for 8 h (E-7) or 12 h (EV-A71, CV-A6, CV-A16) before the culture supernatants were harvested for plaque assays. In pre-treatment assays, RD cells were incubated with compounds in DMEM/F-12 + 2% FBS for 2 h followed by washing with PBS prior to infection. In post-treatment assays, cells were incubated in DMEM/F-12 + 2% containing compounds after virus had been removed and cells washed.

### Compounds

All novel compounds tested was design, synthesized and provided by Curovir AB, G197 is a novel capsid compound bearing structural similarity to molecules published by another group^12^, and BTA-798 (Fig. 1a). All the other mentioned compounds are novel PI4KIIIβ inhibitors (described in patent applications US9963455, US10407429, EP3606926) and N373 bears the base structure shown in Fig. 1b. Compounds were dissolved in DMSO at 20 mg/ml stock concentrations prior to use for all in vitro and in vivo experiments.

### Cell viability assay

Monolayers of RD cells in 96-well format were incubated with compounds dissolved in DMEM/F-12 + 2% FBS for 12 h, after which the cells were washed with PBS and incubated in 10% alamarBlue Cell Viability Reagent in DMEM/F-12 + 2% FBS. Triplicate wells were then read at 570 nm and 600 nm (normalization) and cell viabilities were expressed as a percentage with reference to control, 0.1% DMSO treated cells, as 100%.

### Viral plaque assay

Monolayers of RD cells in in 24-well plates were washed and incubated with 100 µl of tenfold serially diluted culture supernatants for 1 h at 37 °C. Virus was removed and the cells were washed with PBS then overlaid with 0.1% agarose in DMEM/F-12 + 2% FBS. Wells were fixed with 4% formaldehyde and stained with crystal violet before the plaques were counted and expressed as plaque forming units per millilitre (PFU/ml).

### Time-of-addition assay

Virus inoculation was performed from − 1 to 0 h post-infection and cells were washed prior to addition to virus and after removal of virus. For cells that were treated prior to virus inoculation, fresh compounds were added during virus inoculation and at 0 h after virus had been removed. Infected cells were incubated at 37 °C, 5% CO_2_ until 12 h.p.i. and the culture supernatants were harvested for viral plaque assay.

### Virus thermal stability assay

Virus suspensions were prepared with media supplemented with 2% FBS and 1 µM G197 or 0.1% DMSO at a concentration of 4 × 10^7^ PFU/ml. 100 µl aliquots of each suspension were transferred into sterile, thin-walled PCR tubes and incubated at each temperature for 2 min followed by cooling to 4 °C in a conventional PCR machine. The temperatures used were: 37 °C, 40 °C, 45 °C, 50 °C, 55 °C, 60 °C.

### In vitro PI4KIIIα and PI4KIIIβ activity profiling

Kinase profiling assays were performed in the presence of threefold dilutions of N373 by Reaction Biology Corporation using the ADP-Glo Kinase Assay Kit (Promega) at a concentration of 10 µM ATP. Percentage enzyme activity relative to DMSO control was calculated and curve fits were performed with Prism software (sigmoidal dose–response).

### Murine model of infection

6 day-old Balb/c neonates were infected with 2 × 10^7^ PFU EV-A71 via intraperitoneal injection, treated with compounds dissolved in PBS and observed for 14 days for the appearance of clinical signs and mice were sacrificed at humane end-point based on a clinical scoring chart from previous work by Sun et al.^37^ Harvesting of limb samples for histology and viral load assays were performed at 5 days post-infection (DPI). Samples for histology were fixed in 4% formaldehyde and those for viral load assays were homogenized in PBS and the tissue homogenates were clarified by centrifugation to remove debris. Homogenates were used for viral plaque assays and viral loads were expressed in PFU per 100 mg of tissue.

## Supplementary Information

Supplementary Figures.

## Data Availability

The datasets generated during and/or analyzed in this study are available from the corresponding author on reasonable request.
