# Supplementary material for: Novel capsid binder and PI4KIIIbeta inhibitors for EV-A71 replication inhibition
Source: Sci Rep. 2021 May 6;11:9719. doi: 10.1038/s41598-021-89271-8 (PMC8102518; doi:10.1038/s41598-021-89271-8)
Supplement: Supplementary file 1 — Supplementary Figures. [file 41598_2021_89271_MOESM1_ESM.pdf]

WB antibody: VP2

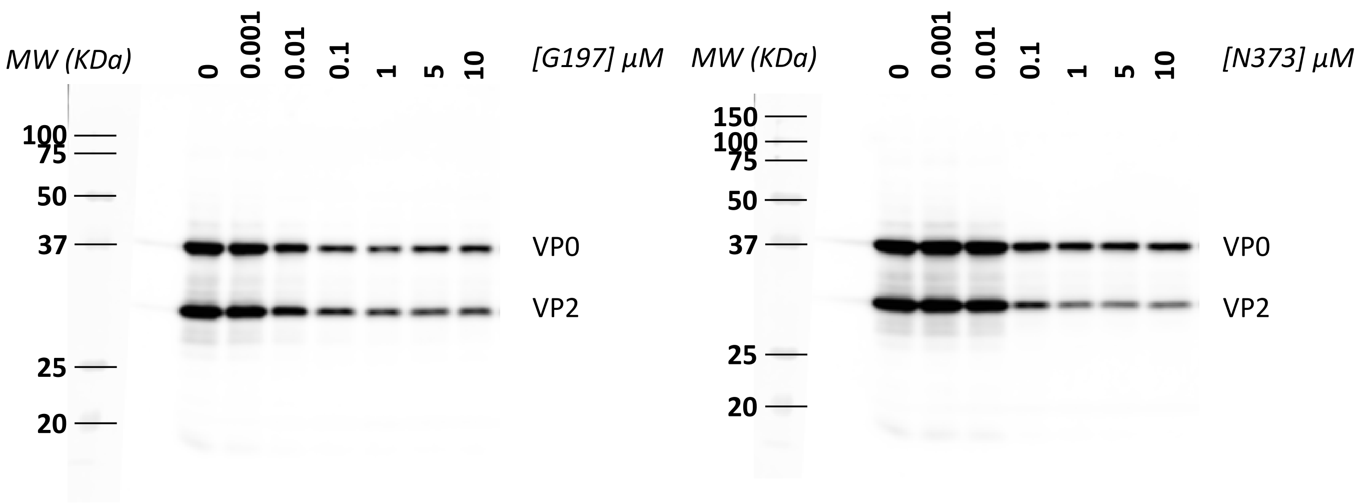

WB antibody: β-tubulin

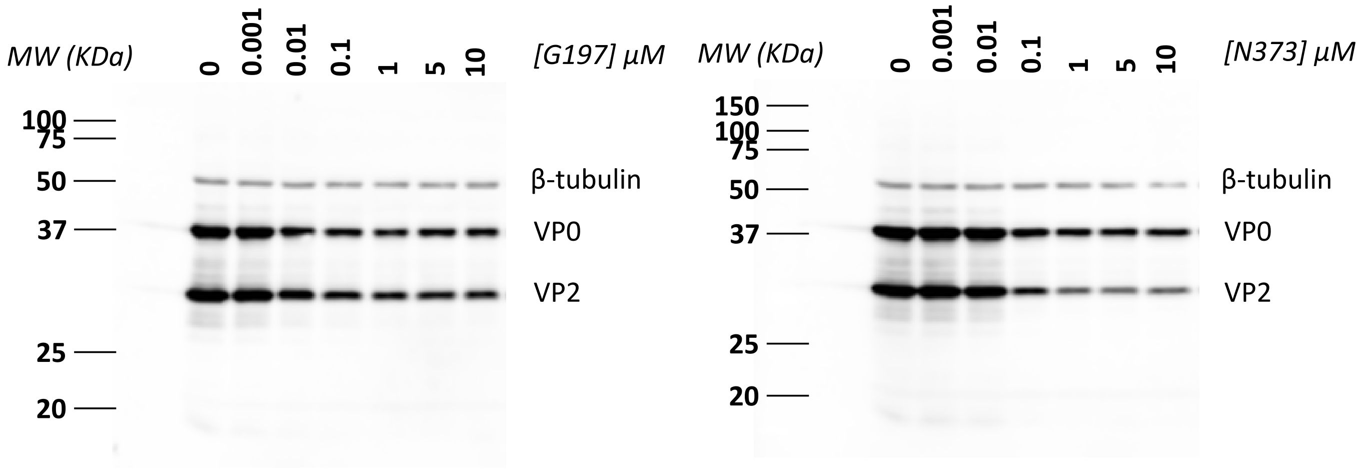

Supplementary Information 1: Original images for Figure 3A and B showing full length of the blots. Concentration 0.001 µM was removed from presented figures to be consistent with the range for viral plaque assays in Figure 2. Membranes were probed with an antibody against VP2 followed by an antibody against β-tubulin without stripping the membranes, hence the bands for VP2 and VP0 were still clearly visible.

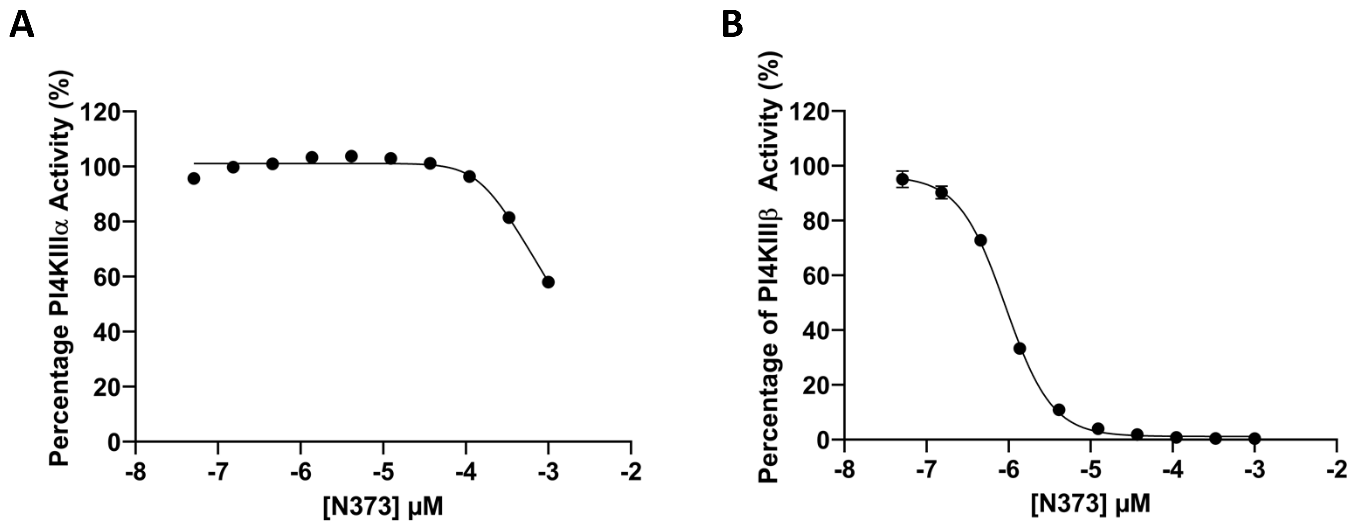

Supplementary Figure 1 Activity PI4KIIIα and PI4KIIIβ at different concentrations of N373.
Activities of **(A)** PI4KIIIα and **(B)** PI4KIIIβ were assessed in the presence of N373 with an ADP-GloTM Kinase Assay (Promega). Luminescence readings were expressed as a percentage activity compared to DMSO controls.

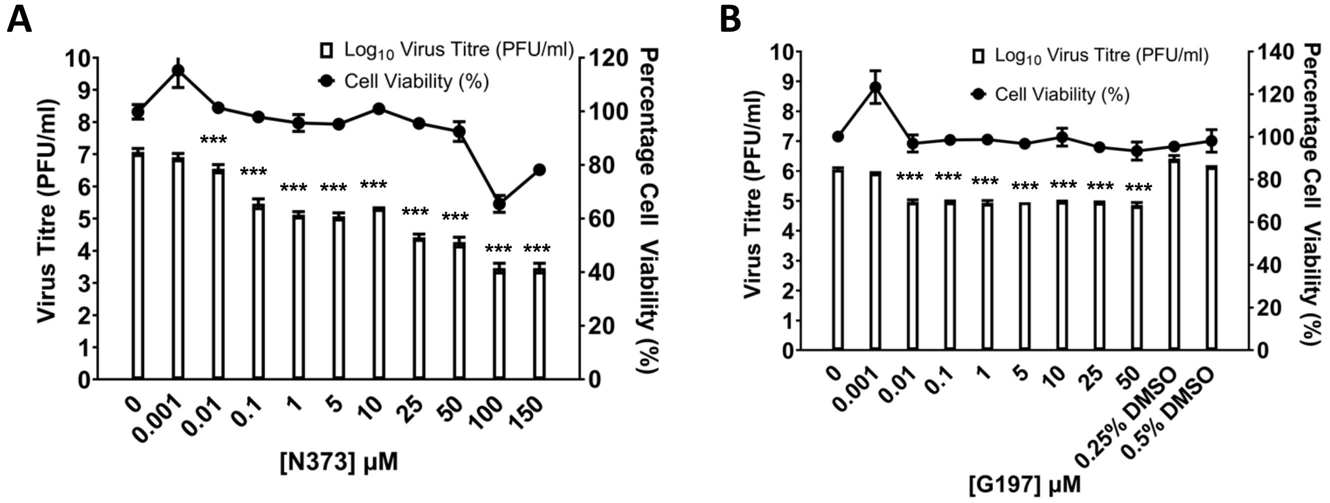

Supplementary Figure 2 Higher concentrations of N373 resulted in a greater inhibition of EV-A71 replication but not for G197.

(A) Virus titres of EV-A71 at concentrations of N373 beyond 10 μM were reduced further and obvious cytotoxicity (viability < 80%) was observed at concentrations from 100 μM. **(B)** Treatment of EV-A71 infected cells with G197 above 10 μM did not result in a greater reduction in virus titres achieved. Statistical analysis for differences in virus titre due to drug treatment was performed with one-way ANOVA with Dunnett's post-test: *** ($p < 0.005$). Concentrations of G197 at 25 μM and 50 μM has higher DMSO content hence additional controls of 0.25% and 0.5% DMSO treatment were added. All other concentrations of N373 and G197 were compared to 0 μM (0.1% DMSO).
